# Application of a Gyroid Structure for Thermal Insulation in Building Construction

**DOI:** 10.3390/ma17246301

**Published:** 2024-12-23

**Authors:** Beata Anwajler, Jerzy Szołomicki, Paweł Noszczyk

**Affiliations:** 1 Faculty of Mechanical and Power Engineering, Wroclaw University of Science and Technology, 50-370 Wroclaw, Poland; beata.anwajler@pwr.edu.pl; 2 Faculty of Civil Engineering, Wroclaw University of Science and Technology, 50-370 Wroclaw, Poland; pawel.noszczyk@pwr.edu.pl

**Keywords:** 3D printing, thermal insulation, gyroid structure, energy audit, energy-efficient building, thermal conductivity

## Abstract

This paper concerns research into the use of 3D-printed gyroid structures as a modern thermal insulation material in construction. The study focuses on the analysis of open-cell gyroid structures and their effectiveness in insulating external building envelopes. Gyroid composite samples produced using DLP 3D-printing technology were tested to determine key parameters such as thermal conductivity (λ), thermal resistance (R) and heat transfer coefficient (U) according to ISO 9869-1:2014. In addition, the authors carried out a comprehensive analysis of the annual energy balance of four different residential buildings, including older and modern structures, using Arcadia software v9.0. The results showed that 100 mm-thick multi-layer gyroid structures achieve exceptionally low thermal conductivity (approximately 0.023 W/(m·K)), significantly outperforming traditional materials such as mineral wool or polystyrene foam in terms of insulation efficiency. These structures also have high mechanical strength and low density, making them both lightweight and highly durable. As a result of these properties, the structures studied represent a promising solution for designing energy-efficient buildings, effectively reducing heating energy demand and improv the overall energy balance of buildings.

## 1. Introduction

In the global construction industry, sustainable development policies require buildings to minimize environmental impact and maximize energy efficiency throughout their lifecycle [1,2]. Among the many parameters that determine the energy efficiency of a building, thermal insulation plays a key role. The thermal insulation of modern building envelopes depends primarily on the insulation properties and thickness of the insulation material [3]. When selecting the appropriate thermal insulation material, both in new projects and in the modernization of existing buildings, it is worth paying attention to parameters such as thermal conductivity coefficient λ, bulk density, acoustic insulation, water vapor permeability, diffusion resistance coefficient μ and resistance to biological, chemical, and fire factors. Buildings lose approximately 35% of their heat through the exterior walls and 25% through the roof, prompting research into new materials and geometric solutions that could reduce heat exchange between walls in contact with the outside air. Current research focuses on the use of 3D printing to design new configurations of exterior partitions with complex biomimetic structures that are lightweight and highly durable [4,5,6,7,8]. Various geometric patterns are used for analysis, such as symmetry, Schwarz-D, Schwarz-P [9], lattice, Hilbert curve [10], linear and rectangular arrangements, hexagonal, as well as honeycomb [11] and spiral structures. These topologies offer diverse thermal and mechanical properties, allowing design engineers to tailor solutions for specific applications, balancing thermal insulation, mechanical strength, and manufacturing cost. The gyroid structure has attracted significant attention as an insulating material in construction due to its unique spatial geometry, which combines a high surface-to-volume ratio with low density. This structure effectively traps air, enhancing thermal insulation by limiting heat penetration while dispersing airflow through complex pathways. The gyroid’s intricate geometry also acts as a thermal barrier, reducing heat transfer. Despite its lightweight nature, the structure exhibits high resistance to compression, bending, and material fatigue, making it durable under dynamic environmental conditions. Their interconnected and uniformly distributed channels effectively distribute stress, minimizing concentrations that could lead to material failure. This enhances the durability and reliability of these structures. Christopher W. Haney and Hector R. Siller [12] conducted a comprehensive study focusing on the mechanical and energetic properties of gyroid structures. Their research involved fabricating gyroid specimens using advanced 3D printing technology and subjecting them to cyclic compressive loading. This approach allowed the evaluation of key mechanical properties such as Young’s modulus, plateau stress, densification strain, and energetic properties over multiple cycles. The study also investigated the mechanisms underlying the energy absorption capabilities of these structures. The results showed that the gyroid structures have high Young’s modulus, with values dependent on density, and significant energy absorption capacity. These properties make gyroids particularly suitable for structural applications where strength optimization and mass reduction are critical. The ability of gyroid structures to uniformly distribute stresses and efficiently absorb dynamic energy highlights their potential as materials for thermal insulation systems, seismic protection, and structural load-bearing elements. In addition, the inherent geometry of the gyroid structure facilitates material reduction, which is consistent with the design principles of energy-efficient and sustainable building practices.

In addition to thermal and mechanical properties, gyroid structures exhibit exceptional acoustic insulation capabilities. The open-cell design allows air to flow through the structure, a critical feature for sound absorption. Porous materials such as gyroids effectively dissipate sound waves, reducing their transmission. The interconnected channels trap sound waves, promoting their dissipation and conversion to heat. In addition, the unique geometry scatters and diffuses sound waves in multiple directions, helping to control reverberation and reduce noise.

The potential of gyroid structures as sound insulators was further investigated by Takumi Yano, Akiko Sugahara, and Yasuhiro Hiraguri [13]. In their study, numerical simulations and experimental methods were used to evaluate the influence of gyroid geometries on sound wave transmission. The research showed that gyroid structures have significant sound insulating properties, with different efficiencies in different frequency ranges. The size of the gyroid structure plays a critical role in its ability to absorb or block sound waves, further emphasizing the importance of geometric optimization for acoustic applications.

Although the mechanical, thermal, and acoustic advantages of gyroid structures are well-documented, the cost effectiveness of their production compared to conventional materials remains an underexplored area. Svetlana Besklubova and others [14] addressed this gap by investigating the economic implications of using 3D printing (3DP) versus conventional construction methods. Their study developed mathematical models to analyse the costs associated with various production factors, including research and development, architecture, engineering, transportation, installation, printing, assembly, and waste disposal. Real-world case studies were used to validate these models. The results indicate that traditional methods may still have an economic advantage for large-scale construction projects, mainly due to established economies of scale. However, on-site 3D printing shows significant potential to improve cost efficiency by reducing labour and material waste, especially for projects requiring custom components or intricate designs. In addition, the research highlighted that 3D printing enables the precise manufacture of geometrically complex structures, such as gyroids, which are difficult to produce using conventional methods. This capability reduces the need for additional tooling, further lowering costs in scenarios requiring highly intricate or customized designs. In addition, the reduction in material waste is particularly relevant to sustainable construction practices, minimizing environmental impact and aligning with circular economy principles. The economic feasibility of gyroid structures also depends on the scalability of 3D printing technologies. Current advances in additive manufacturing are beginning to overcome limitations in production speed and material availability. As these technologies mature, the per-unit cost of 3D-printed components is expected to decrease, making them increasingly competitive with traditional materials. In addition, on-site 3D printing eliminates the cost of shipping prefabricated parts, which can be significant for large or remote projects.

## 2. Review Literature

In recent years, extensive research has focused on the use of 3D printing to improve the thermal efficiency of building envelopes through topological modifications. Sarakinioti et al. [15] developed a facade panel that integrates insulation properties with thermal storage in a complex monomaterial geometry created using 3DP. Mihalache et al. [16] studied the effect of complex interior wall structures on thermal performance to prevent overheating due to thermal radiation. Grabowska and Kasperski [17] proposed the use of 3DP to produce prototypes of plastic insulation materials. They designed and printed multilayer materials in the form of quadrangles, hexagons, and triangles. They later developed a mathematical model and validated it through experiments to identify the most efficient configuration. Dey et al. [18] analysed the thermal performance of 3D-printed concrete slabs that could potentially be used as walls. The slabs are designed with hollow interiors to accommodate insulation material. Kaszynka et al. [19] compared the performance of traditional walls with 3D-printed walls. Their research showed that the corrugated structure of the 3D-printed walls increased mortar adhesion, resulting in effective thermal insulation attachment. Alkhalidi et al. [20] analysed the optimal void layout in 3D-printed concrete facade elements. Maraias et al. [21] evaluated the thermal performance of printed concrete walls with microstructured voids to determine the most efficient geometry by studying the effect of void number and size on thermal transmittance. Nemova et al. [22] conducted theoretical and experimental studies on the thermal performance of 3D-printed concrete (3DPC) partitions with different configurations, material arrangements, and insulation types. Suntharalingam et al. [23] studied different topological variants of walls to determine the most energy efficient configurations. In particular, 32 configurations were analysed, both with and without different insulating materials. Cuevas et al. [24] studied seven configurations using two different cement mixes. He et al. [25] designed a prototype building envelope, 3D-VtGW, consisting of 3D-printed modular elements that serve as a framework for a green wall system to improve the energy efficiency of the building. De Rubeis [26] conducted experimental studies on the thermal insulation properties of three polylactic acid (PLA)-printed blocks with multi-row, square, and honeycomb structures, respectively. The results showed that increasing the complexity of the internal structure led to a reduction in heat transfer, which was confirmed by the hexagonal cells. Wallat et al. [27] investigated the design and characterization of gyroid structures for applications requiring tailored porosity. A MATLAB-based procedure was developed to create gyroid structures with adjustable porosity gradients. As a result, the pore size and distribution could be controlled. In this study, a gradient-based approach was used to vary porosity in different sections of the structure. The results showed that by controlling the porosity gradients, it is possible to optimize the gyroid structures for better thermal retention and mechanical stability.

In parallel with the research on thermal insulation of biomimetic structures, research on strength analysis is being conducted. Dongxia Yang et al. [28] conducted a detailed study of honeycomb structures with variable wall thicknesses, focusing on improving mechanical properties such as compressive strength and energy absorption capacity by modifying the thickness of the cell walls. The studies compared different types of honeycomb structures: hexagonal, square, and quasi-square, which had identical Poisson’s ratios but different Poisson’s ratios. The test results showed that structures with a gradient wall thickness had higher strength compared to structures with a uniform wall thickness. Zhao et al. [29] investigated hexagonal and square honeycomb structures under longitudinal and lateral compression, analysing system strength, stiffness, and energy absorption capacity. Optimal cell layout and geometry were found to be crucial in improving structural efficiency, especially in the context of different types of loading. Wang et al. [30] have focused on improving the mechanical properties of honeycomb structures using hybrid materials and optimized geometry. Hybrid honeycombs produced by 3D printing with different cell configurations were used to increase their resistance to dynamic loading and energy absorption capacity. The structures obtained with the addition of composite materials were characterized by greater strength and durability with minimal weight. In addition to research on thermal insulation, energy efficiency, and durability, research is being conducted on functional gradient materials associated with a gradually changing density. Such materials can be used in the exterior walls of buildings exposed to high winds or seismic activity. Zeyrek et al. [31] described the use of composite panels based on a honeycomb structure that can perform structural and thermal insulation functions. The researchers used phase change materials (PCMs) inside the honeycomb cells, which allowed the panels to store and gradually release heat. The experiments carried out included mechanical and thermal tests. The tests included bending the sample to check its structural strength, and heating and cooling cycle tests to analyse the thermal efficiency of the PCM. The results showed that the panels with PCM had significantly better heat release and storage properties compared to structures without PCM. Despite numerous studies on biomimetic structures, the scientific literature lacks practical analyses of the possibilities of their application in construction and comparison with traditional solutions by performing an energy balance.

The purpose of this study was to design, fabricate, and determine the thermal properties of open-cell 3D composites with an internal gyroid core structure. The study does not focus on the mechanical, acoustic, or economic aspects. The research then analysed the impact of using 3D-printed thermal insulation on reducing the energy required to heat a building over the course of a year. This analysis was conducted using the ArCadia program (case study). This software is used to determine the energy demand of a building according to the EN ISO 13790 standard [32] by balancing the energy demand in the building on a monthly basis (losses and gains). The presented research not only enhances the understanding of the potential of gyroid structures in thermal insulation, but also demonstrates their practical application in energy-efficient construction, as illustrated by a real case study. By bridging the gap between theoretical research and practical implementation, this study highlights the versatility and scalability of gyroid structures as a sustainable solution for construction.

## 3. Materials and Methods

### 3.1. Design and 3D Printing of Gyroidal Structure

Based on previous research by the co-author of this article [33,34], composites with gyroid-shaped internal core structure geometries produced using DLP 3D printing technology were selected for analysis. DLP printing technology is a process similar to SLA, except that the resin is cured not by a laser beam, but by light emitted from a projector mounted beneath the resin reservoir. Recently, several companies have introduced a modification of this technology that replaces the DLP projector with an LCD. With this solution, the entire layer is exposed and cured at the same time, streamlining the printing process.

The gyroid-textured samples were produced on an Elegoo Mars 4 Max printer for layer-by-layer curing of liquid resins. A key feature of this process is its high level of accuracy, allowing even the finest details to be produced with precision.

Research has focused on the gyroid structure. The basic formula describing the gyroid is in the form of Equation (1) [35,36,37].

t = sin (x) cos (y) + sin (y) cos (z) + sin (z) cos (x)(1)

When the equation was entered into the parametric modelling software, a standard TPMS appearance was obtained. However, gyroid structures can be infinitely modified by introducing coefficients into the base equation. In [35], an extension of the gyroid equation with additional variables was proposed, as follows:

(2) $$t=\left[sin\left(\frac{2\pi \cdot x}{a}\right)-cos\left(\frac{2\pi \cdot y}{b}\right)\right]+\left[sin\left(\frac{2\pi \cdot y}{b}\right)-cos\left(\frac{2\pi \cdot z}{c}\right)\right]+\left[sin\left(\frac{2\pi \cdot z}{c}\right)-cos\left(\frac{2\pi \cdot x}{a}\right)\right]$$

where a, b, c—the size of the gyroid’s air cells.

The choice of a particular gyroidal structure was dictated by an earlier experimental study conducted by one of the co-authors [33]. The research involved determining the thermal properties of gyroidal structures as a function of the cell sizes a, b, and c in the core of the structure and the parameter t, which describes the wall thickness of the gyroid. The prototype insulating partitions with gyroidal structures were then generated that varied in wall thickness t (t = 0.2; 0.6; 1.0 mm) and cell sizes a, b, and c (a, b, c = 2π or a, b = 3π c = 2π or c = 3π a, b = 2π). The prototype tiles had dimensions of 50 mm × 50 mm × 20 mm. Each variant was generated as a three-variant set of the number of structural layers: *n* = 1, 2, 3. Based on a multi-criteria analysis, the optimal composition of the composite was determined according to the adopted optimization criteria. The lowest thermal conductivities were obtained for prototype insulating partitions with a gyroidal structure characterized by wall thickness t = 0.2 mm, cell sizes a, b, and c (with a, b = 3π and c = 2π), number of structural layers n = 3 and cooling from below. The lowest possible thermal conductivity of the insulation was 0.033 W/(m·K) and the highest possible thermal resistance was 0.606 (m^2^·K)/W. The following samples were fabricated for the tests described in this paper.

### 3.2. Geometry of the Samples

**Figure 1:**
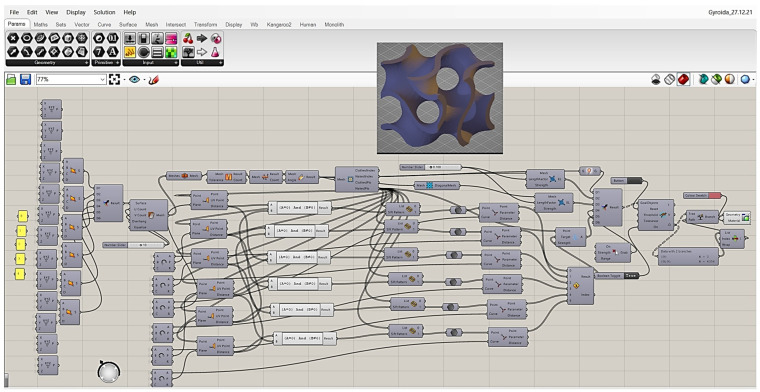
Grasshopper algorithm for the creation of gyroid structures.

Using advanced 3D computer modelling software (Rhino 7), the entire process is based on pregenerated cross-sectional data—Figure 1.

**Figure 2:**
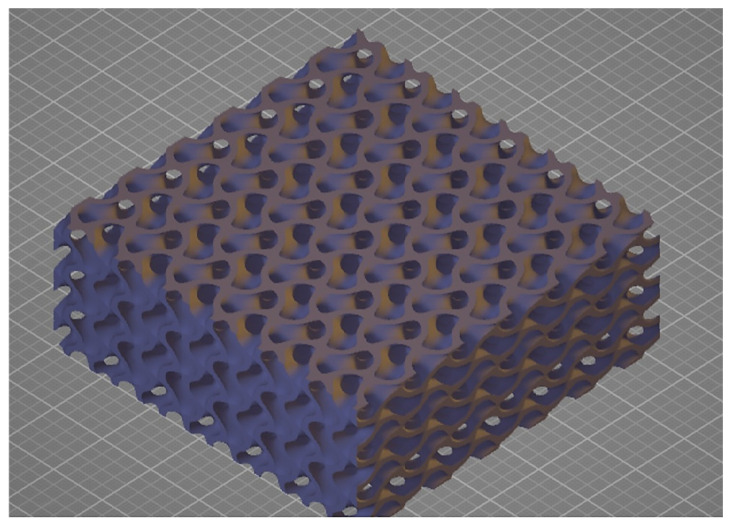
Design of an example of the inner core of a sample with a single layer of material.

The prototype insulating partitions with a gyroidal structure were produced with a wall thickness t (t = 0.2 mm) and internal cell sizes a, b, and c (a, b = 3π, c = 2π) [33]—Figure 2.

**Figure 3:**
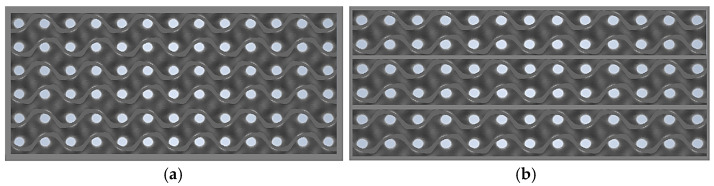
Layering of samples with a thickness of 60 mm: (**a**) one layer and (**b**) three layers.

The DLP process was used to print prototypes of thermal insulation panels with a total insulation thickness of 60 mm (single and triple layer with spacers every 20 mm in the composite), 80 mm (single and four layer with spacers every 20 mm in the composite), and 100 mm (single and five layer with spacers every 20 mm in the composite). A grey UV resin was used for printing. The samples were made with dimensions adapted to the test bench, i.e., 50 mm × 50 mm. A total of six types of samples were made, each of which was repeated three times. A sample design is shown in Figure 3.

The flow of thermal energy through an insulation layer is an extremely complex process. It involves heat conduction in the gaseous and solid components, heat transfer by radiation within the elements, convection, and the movement of moisture in the pores associated with absorption and desorption. The index used to evaluate the effectiveness of thermal insulation, called the effective thermal conductivity coefficient (λ), is highly dependent on factors such as pressure, temperature, chemical composition of solids and gases, porosity, particle shape, dimensions and many other parameters. Insulating materials use mechanisms to minimize the effect of each of these elements on the overall heat transfer to perform their function. A reduction in conduction and convection is usually achieved by using a structure containing many small gas-filled spaces, so layering has been introduced into the open-cell gyroid structure created. This reduces the contribution of convection to the total thickness of the insulation material [33,38]. In turn, radiation reduction is achieved by using low emissivity surfaces that reflect thermal radiation [38,39].

### 3.3. Experiments

The values of the thermal conductivity coefficient (λ), thermal resistance (R) and heat transfer coefficient (U) were experimentally determined for each of the described and printed prototype composite samples. The measurements were performed according to ISO 9869-1:2014 [40] on an existing test rig at the Department of Energy Conversion Engineering, Faculty of Mechanical and Power Engineering, Wrocław University of Technology, described in previous publications by the co-author [33,41,42,43].

The temperatures prevailing on the external side of the sample were chosen on the basis of typical operating conditions of thermal insulation of buildings, i.e., +22 °C (on the ambient side) and −18 °C (in the refrigerator-freezer chamber). The principle of measurement and determination of thermal properties was described in a previous paper by the authors [34].

The accuracy of the measuring instruments is given in Table 1.

## 4. Results and Discussion

### 4.1. Thermal Conductivity, Thermal Resistance, and Transmission Coefficient of Analysed Material Samples

The aim of the study was to evaluate the thermal properties of the printed composites for their potential use as insulation materials according to ISO 9869-1:2014 [40]. Statistical analyses were performed using STATISTICA 13 software (TIBCO Statistica, Palo Alto, CA, USA). A significance level of *p* ≤ 0.05 (a commonly accepted threshold in thermal insulation evaluation) was used. For the results obtained from the experimental testing of the 3D DLP-printed composites, measures of position and dispersion were first calculated, and their results are summarised in Table 2.

**Figure 4:**
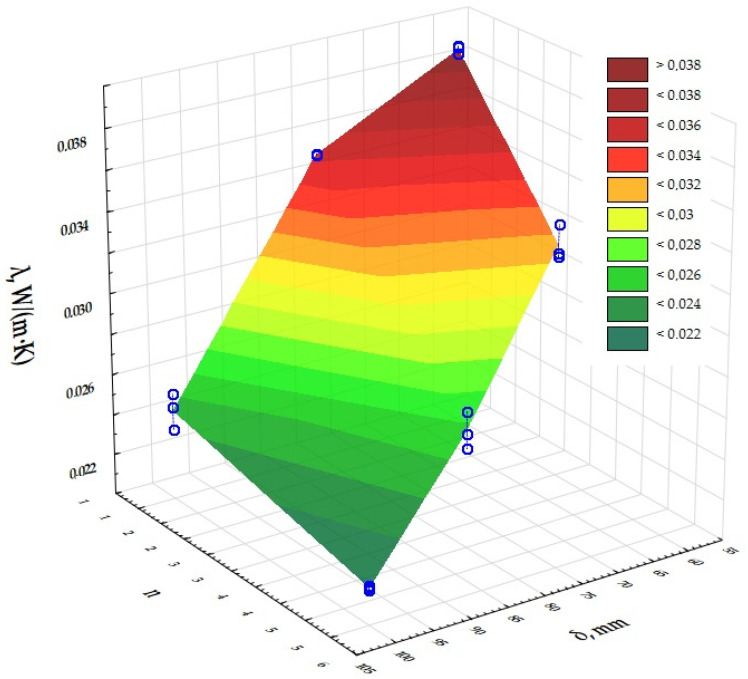
Graphical interpretation of the experimental data determining the influence of input factors (insulation thickness and composite layering) on the value of thermal conductivity of a composite with gyroidal structure.

**Figure 5:**
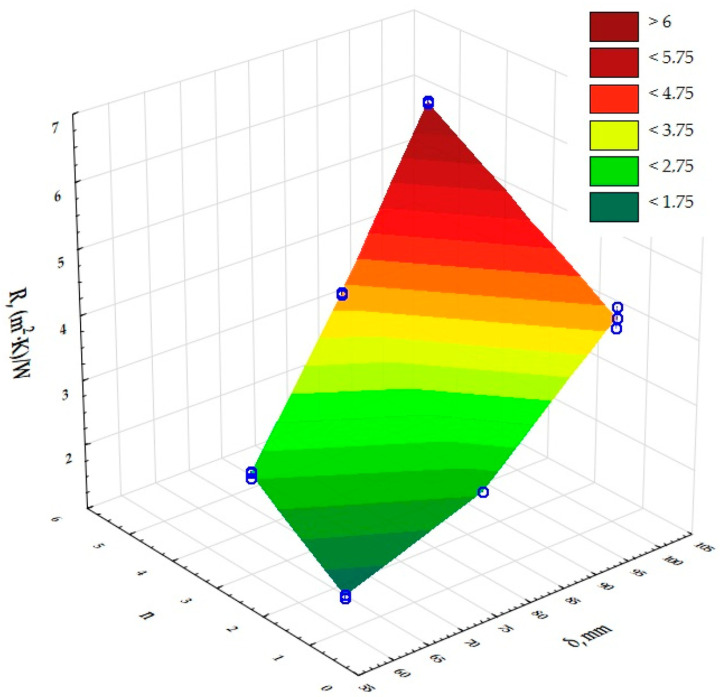
Graphical interpretation of the experimental data determining the influence of input factors (insulation thickness and composite layering) on the value of thermal resistance of a composite with gyroidal structure.

**Figure 6:**
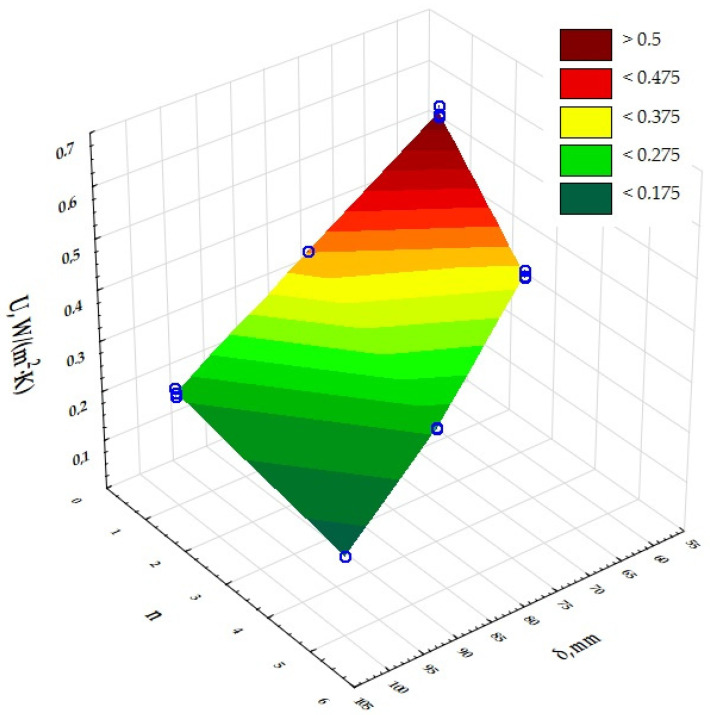
Graphical interpretation of the experimental data determining the influence of input factors (insulation thickness and composite layering) on the value of heat transfer coefficient of a composite with gyroscopic structure.

The next step was to assess the significance of the effect of the size of the independent input variables on the results obtained for the dependent output variables. A two-factor ANOVA analysis of variance was used to determine this effect. The results are presented in Table 3 and Figure 4, Figure 5 and Figure 6. The presented significance level values of *p*, which are less than 0.05, determine the significant influence of the thickness of the composites (δ) and the number of layers in the composite (n) on their thermal conductivity coefficient, thermal resistance and heat transfer coefficient values. The dependence of the thermal conductivity coefficient (λ) on the thickness of the printed composites (δ) and the number of structural layers (n) in the composites was determined. The same was done for the thermal resistance (R) and heat transfer coefficient (U).

Based on a two-factor analysis of variance, a significant effect of the composite thickness (δ) and the number of layers in the composite (n) on the value of the thermal conductivity coefficient, thermal resistance and heat transfer coefficient of the printed composites with a gyroidal inner core structure was demonstrated. It was shown that the thickness of the composites (δ) used in the experiment is a highly dominant factor over the other input factors, and that each input factor can be optimised independently of the others. The lowest thermal conductivities were obtained for the prototype insulating partitions characterised by the number of structural layers n = 5 and a composite thickness of 100 mm. The lowest possible thermal conductivity of the insulation was 0.023 W/(m·K) and therefore the highest possible thermal resistance was 6.089 (m^2^·K)/W and the heat transfer coefficient was 0.159 W/(m^2^·K). According to PN-EN ISO 9229:2020-12 [44], thermal insulation materials achieve a maximum thermal conductivity coefficient of 0.065 W/(m·K). Analysis of the measurements for UV resin-printed composites with a gyroid structure inner core indicates that this composite has comparable thermal properties to composites with an open cell structure with an inner core based on a Kelvin cell produced using the same DLP 3D-printing technology [43], in terms of use as thermal insulation. The heat transfer coefficients for the samples made of grey UV resin with a gyroidal inner core were in the range of 0.16 to 0.20 W/(m^2^·K) for a thickness of 100 mm for single and five layers, while for cell barriers made of different UV resins with a cell diameter of 6 mm and a porosity of 95% they were in the range of 0.14 to 0.24 W/(m^2^·K), including 0.16–0.24 W/(m^2^·K) for the grey resin composite. It can therefore be concluded that the U-values for the open-cell structures tested are comparable and, in addition, have very good thermal insulation parameters. The volume density of the gyroid structures was also determined and ranged from 344.00 to 433.33 kg/m^3^ (0.3 to 0.5 g/cm^3^).

### 4.2. Energy Consumption of a Building—Case Study

The results of thermal conductivity measurements below 0.04 W/(m·K) confirm the high thermal insulation properties of gyroid structures. The thermal conductivity of approximately 0.023 W/(m·K) for 100 mm thick multilayer structures is significantly better (lower) than typical building insulation materials such as mineral wool, polystyrene, and even closed-cell polyurethane (PU) foams. The geometrically stable and relatively hard structure of 3D-printed gyroidal structures can be successfully used as an additional insulation layer in building applications. In this paper, the effect of using 3D-printed gyroidal insulation on the reduction of usable energy demand for heating in a building over a one-year period is analysed. Table 4 compares the thermal conductivity of the tested gyroidal structures with typical building materials (lower value = better thermal insulation material).

#### 4.2.1. Boundary Conditions for Reference Objects

**Figure 7:**
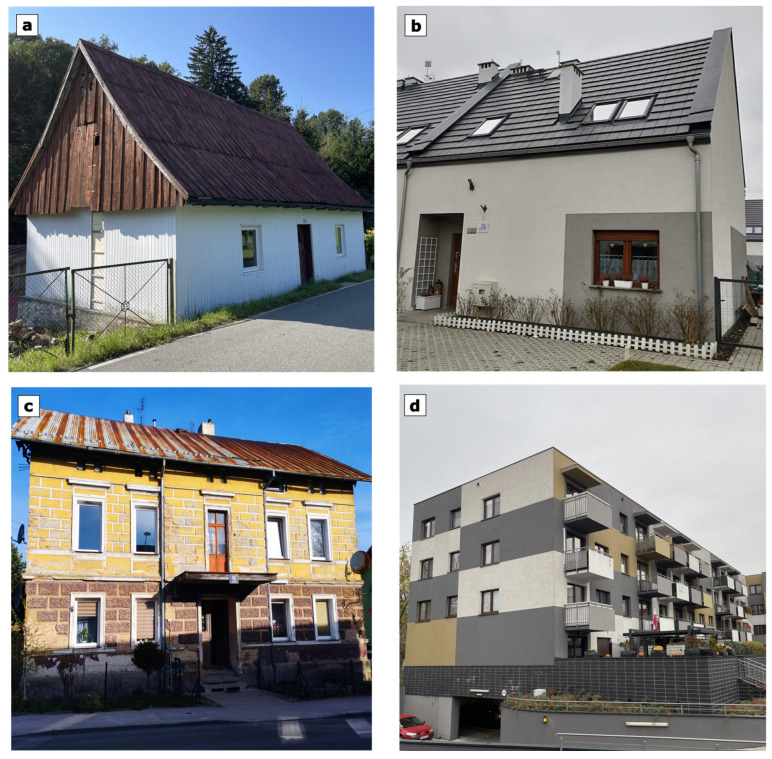
Photographs of the buildings selected for energy analyses: (**a**) old house, (**b**) new house, (**c**) old flat, (**d**) new flat.

**Figure 8:**
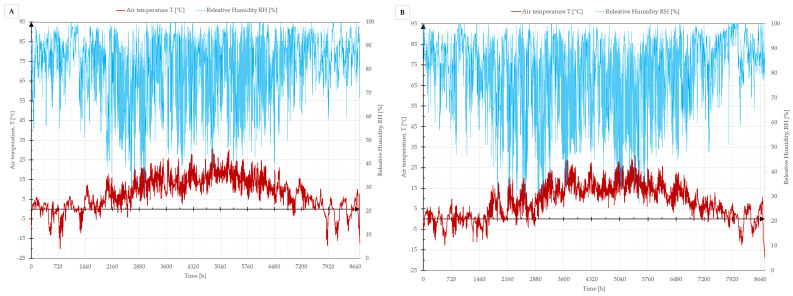
Assumed climatic conditions for the locations considered. The graph shows a typical meteorological year for the cities of Kłodzko (**A**) and Wrocław (**B**).

Four different residential buildings were selected for the analysis, i.e., a house and an apartment in old building (1930s) and new building (2020s) variants; photos of the buildings are shown in Figure 7. The objects selected for the calculations are real buildings located in Lower Silesia in Poland, i.e., in a temperate climate (Figure 8). A temperature of +20 °C was assumed in the heated interior of the building (24 °C for bathrooms) and a typical meteorological year for the nearest weather station was assumed outside. The assumed temperatures of the internal and external environment are in accordance with the EN ISO 13790 standard [32], according to which the methodology for calculating heat losses and gains in a building in a monthly balance was adopted. The internal temperatures were assumed for the whole year (the whole calculation period). In order to simplify the calculations, it was assumed that the buildings were not cooled in summer (which corresponds to reality). Each of the buildings had gravitational ventilation with appropriate assumed ventilation flow coefficients as for residential buildings with continuous ventilation. Other building data, such as building area, ventilated cubic capacity and heat transfer coefficient of individual partitions, can be found in Table 4. Linear heat transfer coefficients according to EN ISO 14683:2017 [46] were used for the calculation of heat losses through thermal bridges, and other data for the buildings are given in Table 5.

#### 4.2.2. Geometry of Building Partitions

**Figure 9:**
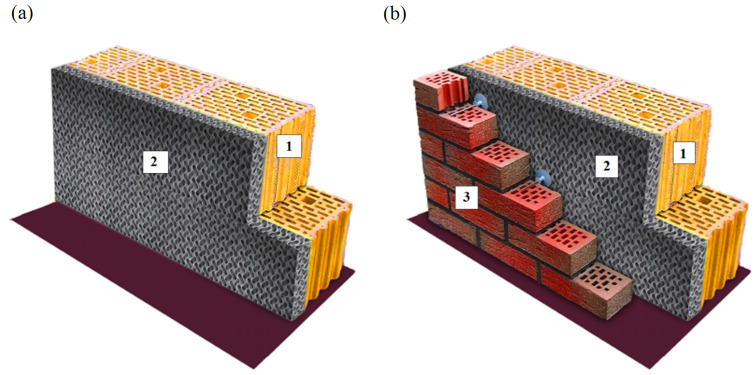
Visualisation of a cross-section of an external wall insulated with 3D-printed gyroidal insulation: (**a**) two-layer wall, (**b**) three-layer wall. Markings: 1—Masonry load-bearing elements—ceramic, 2—Thermal insulation layer made of 3D-printed gyroid, 3—Covering layer made of perforated brick.

When analysing the change in energy demand for heating buildings, a layer of 3D-printed gyroidal thermal insulation was added to all envelopes that occur between the heated space and the outside air (relative to the real envelopes of the reference object). Seven calculation variants were used for the analyses, the first variant being the reference variant, a real building without gyroidal insulation. The remaining six thermal insulation variants correspond to the analysed structures, as shown in the diagram in Section 3.1. An example of the exterior wall cross-section model with gyroidal insulation applied is shown in Figure 9.

#### 4.2.3. Calculation of Energy Performance

The calculation of the useful energy demand for heating the building in the annual balance was performed according to Polish energy calculation methodology in accordance with the guidelines of European standards [47]. The calculations were performed by energy balance of the building in each month separately, taking into account losses through partitions and ventilation, as well as solar and internal gains. The result of the calculations was the usable energy demand for heating and ventilation for the entire year in relation to the whole object, as well as the unit demand per 1 m^2^ of heated area. The obtained results are presented in Table 6.

**Figure 10:**
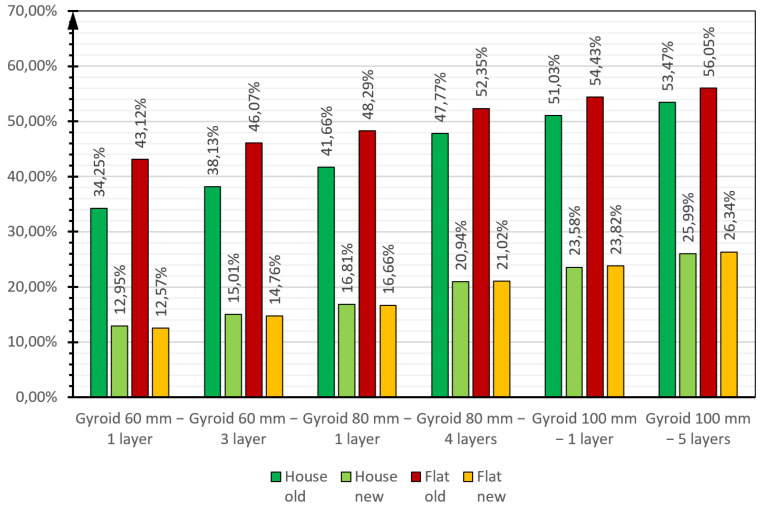
Percentage reduction in usable energy demand in relation to reference buildings (existing building) for each variant of thickness and layering of the proposed thermal insulation material.

The use of the 3D-printed gyroidal structure allowed a significant reduction in the heating and ventilation energy demand of the residential buildings analysed. Figure 10 shows the percentage reduction in usable energy demand compared to the reference buildings for each thickness and layering variant of the proposed thermal insulation material.

The greatest savings in terms of reduction of useful energy demand were obtained for old buildings whose partitions are currently (in the reference building) not insulated. For an old building, the reduction in heating energy demand ranged from 34% for a single-layer 60 mm-thick gypsum board to over 53% for a 5-layer 100 mm-thick gypsum board; for an old apartment, the savings were even higher, ranging from 43% to over 56%. In the case of relatively new buildings (a few years old), the reduction in usable energy demand for heating ranges for the building from almost 13% (60 mm-thick single-layer gyroid) to almost 26% (100 mm-thick 5-layer gyroid), and for the apartment analogously from about 12% to over 26%. These are significant energy savings that make a real difference to the building’s maintenance costs and have a positive impact on the climate.

## 5. Conclusions

The article discusses contemporary solutions used in the thermal insulation of buildings, emphasizing their importance in improving energy efficiency and reducing heating energy consumption. Given the need to reduce heat loss, especially through walls and roofs, the authors highlight the advantages of structures with complex topologies, especially those inspired by biomimetics. In the context of sustainable development, the use of 3D printing to create gyroid structures for thermal insulation was presented, which not only demonstrate high thermal efficiency, but also sound absorption capacity and mechanical strength. Due to their complex geometry, gyroid structures provide excellent insulating properties while maintaining low weight and resistance to external factors. Their ability to retain air, create thermal barriers, and dampen sound makes them an effective thermal insulation material that can replace traditional solutions such as mineral wool or polystyrene. Studies have shown that multilayer gyroid structures achieve a thermal conductivity coefficient of less than 0.04 W/mK, indicating very good thermal insulation properties, better than most standard materials used in building construction.

The research analysed the impact of using thermal insulation from gyroids on the annual energy demand of buildings. The use of this technology allows for a significant reduction in thermal energy losses and an increase in energy efficiency. The results indicate that gyroid structures can significantly reduce heating energy demand, which is especially important in older buildings that are less energy-efficient.

After conducting the research, the authors drew the following conclusions:

- The values of the thermal conductivity coefficient (λ) of all tested samples were in the range from 0.023 to 0.039 W/(m·K), which is lower than the maximum value (0.065 W/(m·K)) required for insulation materials according to PN-EN ISO 9229:2020-12 [44]. This means that the resulting compounds meet the standards of thermal efficiency.
- The input variable of composite thickness (δ) proved to be a highly dominant factor over the other input factors.
- A major advantage of 3D printing that improves the thermal properties of the manufactured cellular composites is the ability to integrate the gyroidal structure with internal spacers in the composite.
- Multilayer gyroid structures are characterized by higher thermal insulation (lower thermal conductivity coefficient) compared to single-layer structures.
- The two-factor analysis of variance (ANOVA) revealed a significant effect of composite thickness (δ) and number of layers (n) on thermal conductivity coefficient (λ), thermal resistance (R) and heat transfer coefficient (U).
- The best insulation results were obtained for composites with a thickness of 100 mm and five layers, where the thermal conductivity coefficient was the lowest (0.023 W/(m·K)) and the thermal resistance the highest (6.089 (m^2^·K)/W).
- Gyroid structures produced by 3D printing exhibit excellent thermal insulation properties, making them a potential replacement for traditional insulating materials.
- Modern structures inspired by biomimetics can effectively support sustainable development goals by reducing heat loss in buildings and reducing the carbon footprint.
- Due to the possibility of modifying structural parameters, such as layer thickness and geometry, they can be tailored to the specific needs of construction, ensuring a balance between energy efficiency and production costs.
- The structures analysed demonstrate high durability and mechanical resistance, which allows them to be used in complex, multi-layer insulation systems that increase the durability and stability of buildings.
- Using 3D-printed gyroid thermal insulation for thermal modernization of buildings, it is possible to reduce the demand for usable energy for heating by more than 50% for old buildings and by about 25% for new buildings, which will significantly improve the energy standard of buildings.

In conclusion, gyroid structures offer an innovative and ecological approach to thermal insulation in construction, and their versatile properties make them a promising material for widespread use in modern and modernized buildings. However, at the current stage of research, their use has certain limitations, especially in the context of real fire conditions [48,49].

The performance of gyroid structures is strongly influenced by the materials used in their construction. Polymers commonly used in gyroid designs can experience a significant loss of mechanical integrity when exposed to elevated temperatures. Although gyroid structures can be designed for applications such as thermal insulation, their inherent open-cell design can facilitate heat transfer, potentially exacerbating the spread of fire. In fire scenarios, this combination of thermal expansion, reduced material strength, and potential deformation poses a risk to the structural integrity of components based on gyroid structures. To address these challenges and improve the fire resistance of gyroid-based systems, future research should focus on several critical areas. First, the application of fire-resistant coatings or the use of inherently fire-resistant materials in gyroid designs should be explored. Such modifications could improve the thermal stability and fire performance of these structures. In addition, detailed thermal analysis and comprehensive fire testing are essential to better understand the behaviour of gyroid structures under extreme conditions and to accurately model their performance. Another important avenue of investigation is the role of geometric parameters, such as cell size and wall thickness, in determining the thermal resistance of gyroid structures. Optimizing these factors could help balance mechanical strength and thermal stability. In addition, the development of hybrid gyroid systems that combine fire-resistant outer layers with lightweight core structures offers promising potential for improved performance in fire-prone environments.

The planned directions of further research include experimental studies of the actual energy efficiency of entire building partitions on a real scale under various temperature conditions simulated in climatic chambers. These research directions are made possible by the availability of unique equipment in the form of advanced climatic chambers.

## Figures and Tables

**Table 1:** The accuracy of the measuring instruments.

Measuring Device	Accuracy
K-type thermocouple	0.1 K
FHF04SC heat flux sensor	11 μV/(W/m^2^)
Vernier caliper	0.05 mm

**Table 2:** Descriptive statistics of thermal conductivity (λ), thermal resistance (R) and heat transfer coefficient (U) (Max—maximum; Min—minimum; Me—median; M—mean; SD—standard deviation; K—curvature; Sk—slant) for all samples produced using 3D DLP printing technology.

	Max	Min	Me	M	SD	K	Sk
λ [W/(m·K)]	0.039	0.023	0.029	0.029	0.006	−1.530	0.229
R [(m^2^·K)/W]	6.089	1.493	3.287	3.457	1.558	−0.892	0.477
U [W/(m^2^·K)]	0.587	0.159	0.298	0.326	0.143	−0.691	0.641

**Table 3:** Quantitative assessment of the main effects—identification of the impact of dominant and statistically significant input factors on the dependent variable λ, R and U (df—degrees of freedom, SS—sum-of-squares, MS—mean square, F—F ratio, *p*—significance level (*p*-values)).

Symbol That Identifies the Input Factors	df	SS	MS	F	*p*
**λ, W/(m·K)**					
absolute term	1	0.0113	0.0113	27,944.61	0.000
δ	2	0.00034	0.00017	418.43	0.000
n	3	0.00023	0.000075	185.97	0.000
error	12	0.000005	0.000		
general	17	0.00068			
**R, (m^2^·K)/W**					
absolute term	1	214.412	214.42	40,936.13	0.000
δ	2	10.892	5.4460	1039.77	0.000
n	3	12.4147	4.1382	790.08	0.000
error	12	0.0629	0.0052		
general	17	41.2495			
**U, W/(m^2^·K)**					
absolute term	1	1.1953	1.1953	28,176.98	0.000
δ	2	0.1782	0.08912	2100.80	0.000
n	3	0.1196	0.0399	939.48	0.000
error	12	0.00051	0.000042		
general	17	0.34643			

**Table 4:** Thermal conductivity coefficient for typical building materials and the analysed 3D-printed gyroid structure (based on standard EN ISO 6946:2017 [45]).

Material	Thermal Conductivity [W/(m·K)]
**Reinforced concrete**	1.70
**Solid brick**	0.77
**Wood**	0.16
**EPS (expanded polystyrene)**	0.040
**Gyroid 60 mm (non-layered)**	0.0393
**Gyroid 80 mm (non-layered)**	0.0353
**Mineral wool**	0.035
**Gyroid 60 mm (layered)**	0.0316
**Gyroid 100 mm (non-layered)**	0.0244
**Gyroid 80 mm (layered)**	0.0244
**PUR (closed-cell)**	0.022
**Gyroid 100 mm (layered)**	0.0200
**Aerogel**	0.014
**VIP (Vacuum Insulation Panels)**	0.006

**Table 5:** Basic boundary data of the energy simulations for the adopted buildings.

Parameter	Unit	Old House	New House	Old Flat	New Flat
**Year of construction**	[-]	1937	2019	1930	2016
**Heated volume**	[m^3^]	143.6	260.4	157.6	142.3
**Heated area**	[m^2^]	61.5	104.0	59.0	54.3
**Ventilation type**	[-]	gravity	gravity	gravity	gravity
**Weather** **station (city)**	[-]	Kłodzko	Wrocław	Kłodzko	Wrocław
**Uc (External wall)**	[W/(m^2^K)]	0.36	0.21	1.17	0.24
**Uc (Windows)**	[W/(m^2^K)]	1.50	0.90	1.10	1.10
**Uc (Internal ceiling)**	[W/(m^2^K)]	0.67	0.18	n.a.	n.a.
**Uc (Ground floor)**	[W/(m^2^K)]	1.26	0.29	n.a.	n.a.
**Uc (Roof)**	[W/(m^2^K)]	n.a.	0.12	0.25	0.19

**Table 6:** Results of usable energy demand for heating and ventilation for the reference and six thermal insulation variants.

		OLD HOUSE		NEW HOUSE		OLD FLAT		NEW FLAT	
Material	Number of Layer	Qh1 [kWh]	Qh1,A [kWh/m^2^]	Qh2 [kWh]	Qh2,A [kWh/m^2^]	Qh3 [kWh]	Qh3,A [kWh/m^2^]	Qh4 [kWh]	Qh4,A [kWh/m^2^]
**Reference**	n.a.	11,492.7	187.0	4688.2	45.09	16,091.3	272.8	2601.4	47.9
**Gyroid 60 mm**	1	7556.1	122.9	4080.8	39.3	9153.3	155.2	2274.4	41.9
**Gyroid 60 mm**	3	7110.8	115.7	3984.9	38.3	8678.3	147.1	2217.8	40.8
**Gyroid 80 mm**	1	6704.4	109.1	3900.2	37.5	8321.4	141.1	2167.9	39.9
**Gyroid 80 mm**	4	6001.7	97.6	3707.1	35.6	7667.1	130.0	2054.5	37.8
**Gyroid 100 mm**	1	5628.2	91.6	3582.9	34.5	7332.4	124.3	1981.9	36.5
**Gyroid 100 mm**	5	5347.5	87.0	3470.2	33.4	7071.5	119.9	1916.4	35.3

Qh—Heat demand of the building per year [kWh]; Qh,A—The heat demand of the building per year for 1 m^2^ [kWh/m^2^].

## Data Availability

The original contributions presented in this study are included in the article. Further inquiries can be directed to the corresponding author.
